# TAPAS: An Open-Source Software Package for Translational Neuromodeling and Computational Psychiatry

**DOI:** 10.3389/fpsyt.2021.680811

**Published:** 2021-06-02

**Authors:** Stefan Frässle, Eduardo A. Aponte, Saskia Bollmann, Kay H. Brodersen, Cao T. Do, Olivia K. Harrison, Samuel J. Harrison, Jakob Heinzle, Sandra Iglesias, Lars Kasper, Ekaterina I. Lomakina, Christoph Mathys, Matthias Müller-Schrader, Inês Pereira, Frederike H. Petzschner, Sudhir Raman, Dario Schöbi, Birte Toussaint, Lilian A. Weber, Yu Yao, Klaas E. Stephan

**Affiliations:** ^1^Translational Neuromodeling Unit (TNU), Institute for Biomedical Engineering, University of Zurich and ETH Zurich, Zurich, Switzerland; ^2^Institute for Biomedical Engineering, ETH Zurich and University of Zurich, Zurich, Switzerland; ^3^Centre for Advanced Imaging, The University of Queensland, Brisbane, QLD, Australia; ^4^Athinoula A. Martinos Center for Biomedical Imaging, Massachusetts General Hospital, Charlestown, MA, United States; ^5^Department of Radiology, Harvard Medical School, Charlestown, MA, United States; ^6^Department of Computer Science, ETH Zurich, Zurich, Switzerland; ^7^Nuffield Department of Clinical Neurosciences, University of Oxford, Oxford, United Kingdom; ^8^School of Pharmacy, University of Otago, Dunedin, New Zealand; ^9^Techna Institute, University Health Network, Toronto, ON, Canada; ^10^Interacting Minds Center, Aarhus University, Aarhus, Denmark

**Keywords:** TAPAS, Translational Neuromodeling, Computational psychiatry, Computational psychosomatics, computational assays, open-source, software

## Abstract

Psychiatry faces fundamental challenges with regard to mechanistically guided differential diagnosis, as well as prediction of clinical trajectories and treatment response of individual patients. This has motivated the genesis of two closely intertwined fields: (i) Translational Neuromodeling (TN), which develops “computational assays” for inferring patient-specific disease processes from neuroimaging, electrophysiological, and behavioral data; and (ii) Computational Psychiatry (CP), with the goal of incorporating computational assays into clinical decision making in everyday practice. In order to serve as objective and reliable tools for clinical routine, computational assays require end-to-end pipelines from raw data (input) to clinically useful information (output). While these are yet to be established in clinical practice, individual components of this general end-to-end pipeline are being developed and made openly available for community use. In this paper, we present the **T**ranslational **A**lgorithms for **P**sychiatry-**A**dvancing **S**cience (TAPAS) software package, an open-source collection of building blocks for computational assays in psychiatry. Collectively, the tools in TAPAS presently cover several important aspects of the desired end-to-end pipeline, including: (i) tailored experimental designs and optimization of measurement strategy prior to data acquisition, (ii) quality control during data acquisition, and (iii) artifact correction, statistical inference, and clinical application after data acquisition. Here, we review the different tools within TAPAS and illustrate how these may help provide a deeper understanding of neural and cognitive mechanisms of disease, with the ultimate goal of establishing automatized pipelines for predictions about individual patients. We hope that the openly available tools in TAPAS will contribute to the further development of TN/CP and facilitate the translation of advances in computational neuroscience into clinically relevant computational assays.

## Introduction

Contemporary psychiatry uses disease classifications that are almost entirely based on syndromes (i.e., patterns of symptoms and signs) as defined by the Diagnostic and Statistical Manual of Mental Disorders [DSM; (1)] or the International Classification of Diseases [ICD; (2)]. While these schemes are valuable in that they provide a stratification of mental illness that relates to the subjective phenomenology of patients, they are inherently limited as they do not rest on pathophysiological or aetiological concepts of diseases. As a consequence, clinical labels proposed by DSM or ICD (e.g., schizophrenia or depression) typically have limited predictive validity with regard to clinical trajectories and do not inform the optimal treatment selection in individual patients (3, 4). Furthermore, clinical and scientific evidence suggests that these labels do not describe distinct categorical entities, but rather spectrum disorders that are characterized by substantial heterogeneity and overlap (5–7).

This has motivated novel approaches to advance our understanding of the pathophysiological and psychopathological processes underlying diseases, and to ultimately inform differential diagnosis and treatment prediction in individual patients (8). In addition to the rise of (epi)genetic approaches, advances in computational neuroscience have fueled hopes that it may become possible to establish quantitative diagnostic and prognostic computational tools that significantly improve clinical practice in psychiatry. In particular, mathematical models of neuroimaging data, as obtained using functional magnetic resonance imaging (fMRI) and electro/magnetoencephalography (EEG/MEG), hold great promise as they might offer readouts of the symptom-producing physiological processes underlying brain disorders (9–14). Similarly, advances in computational models of human behavior may enable inference on psychopathological processes at the computational (information-processing) level (15–20).

**Figure 1:**
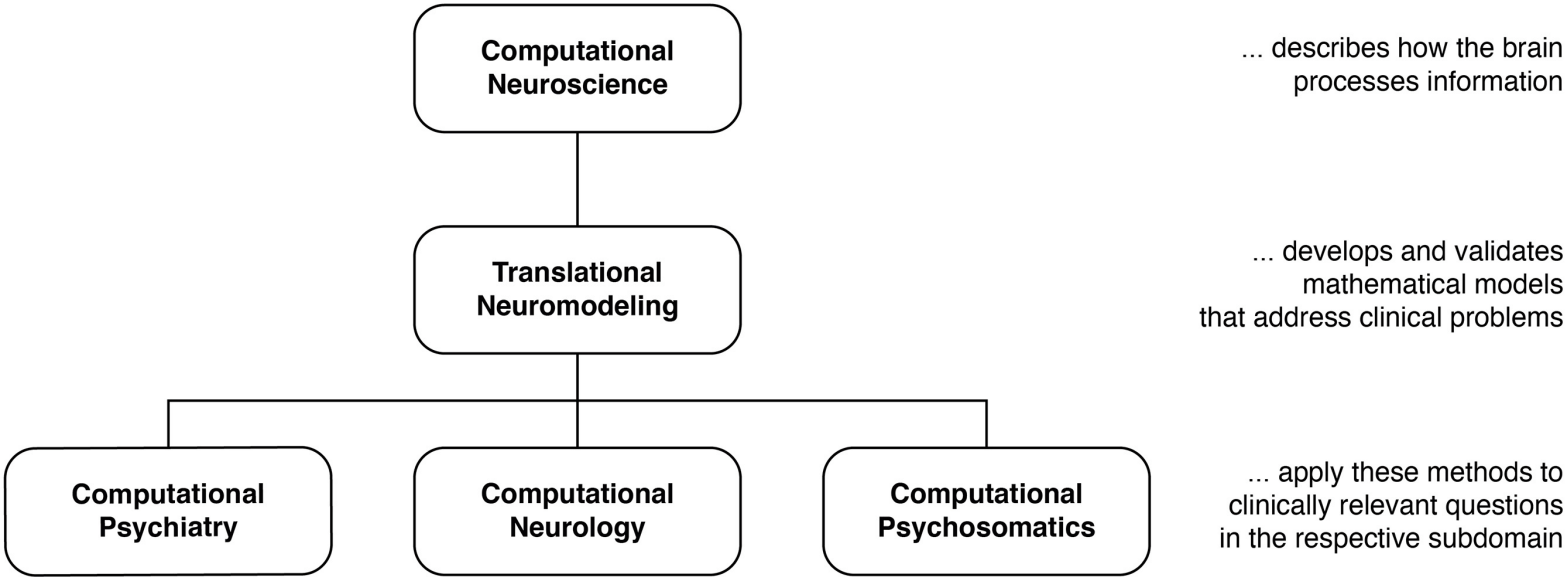
Taxonomy for different disciplines in the computational neurosciences and their relation to clinical questions. Translational Neuromodeling (TN) develops and validates mathematical models for addressing clinical problems, whereas Computational Psychiatry (CP), Neurology (CN), and Psychosomatics (CPS) then apply these methods to clinically relevant questions. Reprinted with permission from Frässle et al. (13). Copyright 2018 Wiley.

Efforts to exploit these scientific advances can be grouped into two separate yet overlapping approaches. Primarily methodological efforts toward the development of “computational assays” for inferring brain disease processes from neuroimaging, electrophysiological, and behavioral data are referred to as Translational Neuromodeling (TN); by contrast, Computational Psychiatry (CP), Computational Neurology (CN), and Computational Psychosomatics (CPS) are concerned with concrete applications in the respective clinical domains, with the ultimate goal of incorporating computational assays into routine clinical decision-making (Figure 1). Although many of the tools in TAPAS are equally useful for CN and CPS, here we focus on CP as this is arguably the most developed of the computational clinical neurosciences (16, 21–29, 114, 119).

Developments of computational assays are often based on generative models of neuroimaging or behavioral data. Generative models describe how measured data may have been caused by a particular (neuronal or cognitive) mechanism; their inversion allows computational assays to operate on inferred states of neural or cognitive systems (16, 29). This mechanistic interpretability is crucial in many clinical contexts. Additionally, traditional machine learning (ML) plays a central role in TN/CP, for example by translating the inferences from computational assays into patient-specific predictions, an approach referred to as “generative embedding” (30).^1^

**Figure 2:**
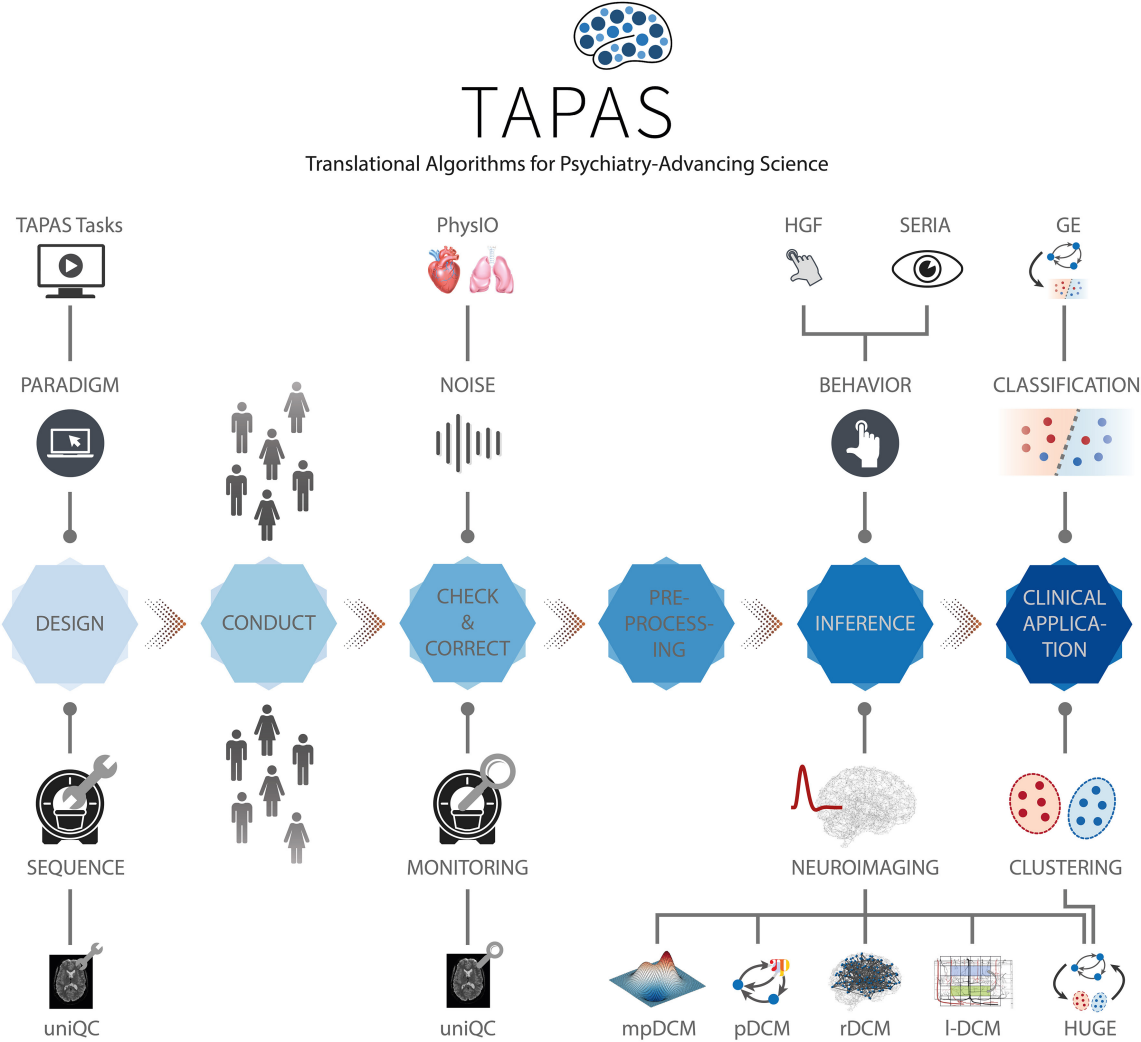
TAPAS components in a proposed end-to-end pipeline of a clinically relevant computational assay. This end-to-end pipeline will need to incorporate all steps from the raw imaging or behavioral data to a final clinical recommendation. Such a computational assays will capture at least the following crucial steps: (i) Design, (ii) Conduct, (iii) Check and Correct, (iv) Preprocessing, (v) Inference, and (vi) Clinical application. Various components of TAPAS feature into one or several of these steps and aim to address important questions and limitations that have so far hampered translational success.

While early TN/CP proposals date back over a decade [e.g., see Stephan et al. (31)], computational assays are yet to enter into routine clinical practice in psychiatry. In order to achieve translational success, computational assays will have to build on automatized and validated end-to-end pipelines and tools for optimal data acquisition. These pipelines need to support a complete analysis stream that takes raw data as input, and outputs clinically actionable results that are derived from inferred latent (hidden) computational quantities with pathophysiological or psychopathological relevance. Such an end-to-end pipeline will incorporate a series of fundamental steps (Figure 2): (i) Design, (ii) Conduct, (iii) Check and Correct, (iv) Preprocessing, (v) Inference, (vi) Clinical application. Each of these components poses significant challenges given the complex nature of the acquired data and of the computational tools. Here, we review the current state of development toward such an end-to-end pipeline, with a particular focus on our own work and software.

Considerable efforts have recently been made to develop standardized and user-friendly software packages that could serve as individual components for computational assays. For instance, in the context of neuroimaging data, packages like Statistical Parametric Mapping [SPM; (32)], FMRIB Software Library [FSL; (33)], or Analysis of Functional NeuroImages [AFNI; (34)] are widespread tools and cover several aspects of the aforementioned end-to-end pipeline, including preprocessing of neuroimaging data and statistical inference (typically in the framework of General Linear Models). Similarly, for behavioral data, software packages like the VBA Toolbox (35), hBayesDM (36), KFAS (37), COMPASS (38), or HDDM (39) allow inference on computational (information processing) quantities. This list is non-exhaustive and more software packages could be mentioned. While all of these packages have proven highly valuable to study behavior and brain function in humans, none of them has been designed with the specific goal of constructing a pipeline for clinically useful computational assays.

In this paper, we focus on the **T**ranslational **A**lgorithms for **P**sychiatry-**A**dvancing **S**cience (TAPAS) software package which represents a collection of toolboxes that, collectively, aim to advance computational modeling of neuroimaging and behavioral data. While applicable to study human behavior and brain function in health, TAPAS differs from the aforementioned software packages in that its designated purpose is to provide clinically useful tools at every stage of the aforementioned end-to-end pipeline in order to advance translational success of computational approaches to psychiatry. TAPAS is primarily written in MATLAB (with some components in C and Python) and distributed as open-source code under the GNU General Public License 3.0 (https://www.translationalneuromodeling.org/tapas). It does not represent a single unified piece of software but rather a collection of toolboxes, each of which addresses a specific problem that arises in TN/CP approaches to neuroimaging and/or behavioral data analysis (Figure 2). More specifically, the development of each toolbox has been motivated by the general goal of TN/CP: to develop end-to-end pipelines that derive clinically actionable outputs from measured data, as illustrated in Figure 2.

In brief, TAPAS contains: (i) tailored experimental paradigms (tasks) that probe psychopathologically and/or pathophysiologically relevant processes, (ii) tools for optimization and monitoring of data quality in the specific context of fMRI, (iii) model-based physiological noise correction techniques for fMRI data, and (iv) generative models and associated statistical techniques that enable inference on latent (hidden) neurophysiological or cognitive quantities from neuroimaging or behavioral data. The latter range from network/circuit models that infer effective (directed) connectivity from fMRI and EEG/MEG data to behavioral models that extract computational quantities from observed actions (e.g., decisions or eye movements). Importantly, TAPAS is not meant to provide a comprehensive collection of all tools that may potentially contribute to the development of end-to-end pipelines for computational assays. Instead, TAPAS represents a collection of toolboxes that are of particular strategic and practical relevance for advancing TN/CP and for supporting translational applications of computational approaches to problems in psychiatry.

To facilitate usability of our software, TAPAS is complemented with comprehensive documentation for each toolbox, as well as an active forum where users can seek help (https://github.com/translationalneuromodeling/tapas/issues).

Here, we provide a general overview of the different software toolboxes included in TAPAS and highlight how these may support the development of clinically useful computational assays for psychiatry. The paper is not meant to provide a comprehensive description of each toolbox, but instead offers a high-level perspective on how the different tools relate to each other in order to jointly advance TN/CP. For readers interested in a more in-depth treatment of a particular toolbox, references will be provided in the respective sections.

## Design

The development of carefully designed experimental manipulations and the acquisition of high-quality data is paramount for (clinical) modeling. This is because any conclusion—whether a scientific or clinical one—fundamentally rests on the underlying data. The goal of tailored experimental paradigms and optimized data acquisition is to increase both the sensitivity and specificity of clinical tests; this necessitates optimizations at different stages of data collection.

*(I) Prior to data collection*: Tailored experimental paradigms have to be designed that capture relevant processes of interest. This may relate to physiological and cognitive aspects in health, or to pathophysiological and psychopathological mechanisms in disease. Furthermore, optimization of the data acquisition process is vital to ensure high quality measurements. This is particularly important in the context of fMRI data, where it is common that project-specific MR sequences have to be developed. These aspects will be covered in the current section.

*(II) During data collection*: Measures have to be taken that allow maintaining a consistently high level of data quality across acquisitions; for instance, across different patients, scanners or sites. This is vital in order to ensure that comparable (clinical) conclusions can be drawn from the data. Tools that address this aspect of data quality control will be covered in section Conduct, Check and Correct.

*(III) After data collection*: *Post-hoc* assessment of data quality is important to identify datasets that need to be excluded or extra analysis steps to deal with artifacts. Poor data quality might be due to severe artifacts and/or high noise levels in the data, induced by both the MR system and the participant itself (motion, physiological noise). To identify such cases, tools are required that allow for quantitative assessment of data quality and that facilitate the decision process as to whether satisfactory data quality can be restored or not. Finally, user-friendly tools are needed that enable automatized corrections to clean-up data as best as possible. We will address these aspects in section Preprocessing.

All these efforts ideally interact seamlessly with each other in order to maximize the sensitivity and specificity of diagnostic/prognostic tests that build on the acquired data. Suboptimal data acquisition and quality control can result in a high proportion of datasets that have to be excluded from further analyses or lead to false conclusions, which is particularly problematic in the context of clinical applications.

### Harmonization of Experimental Design

For probing disease-relevant cognitive processes, a plethora of experimental tasks have been proposed that frequently only differ in small details. While some variations are valuable as they address somewhat different aspects of a cognitive process, this diversity complicates exact comparison of findings across studies and often gives rise to “approximate replications” where an initial finding is not replicated exactly but some (vaguely) related finding is linked to the original observation (4, 40). A prominent example of this in the context of clinical neuroimaging is the frontal dysfunction hypothesis in schizophrenia. Since the original report (41), several studies have re-examined this question using somewhat different experimental approaches and have reported a variety of different outcomes, ranging from hyper- to hypoactivation, to no obvious alterations at all [cf. (4, 42)]. While it is possible that these differences could be of pathophysiological relevance, potentially referring to different subgroups in the schizophrenic spectrum, inconsistencies in the utilized experimental manipulations render any differences difficult to interpret. Hence, until such variations are properly explained or controlled for, approximate replications do not provide a solid basis for clinical tests.

One way to address this challenge is by openly sharing established experimental tasks. Notably, while the call for open sharing of data has been very prominent in recent years (43–45), this is less the case for sharing the experimental tasks themselves (but see, for instance, the task protocols utilized by the Human Connectome Project (46) which have been shared openly).

**Figure 3:**
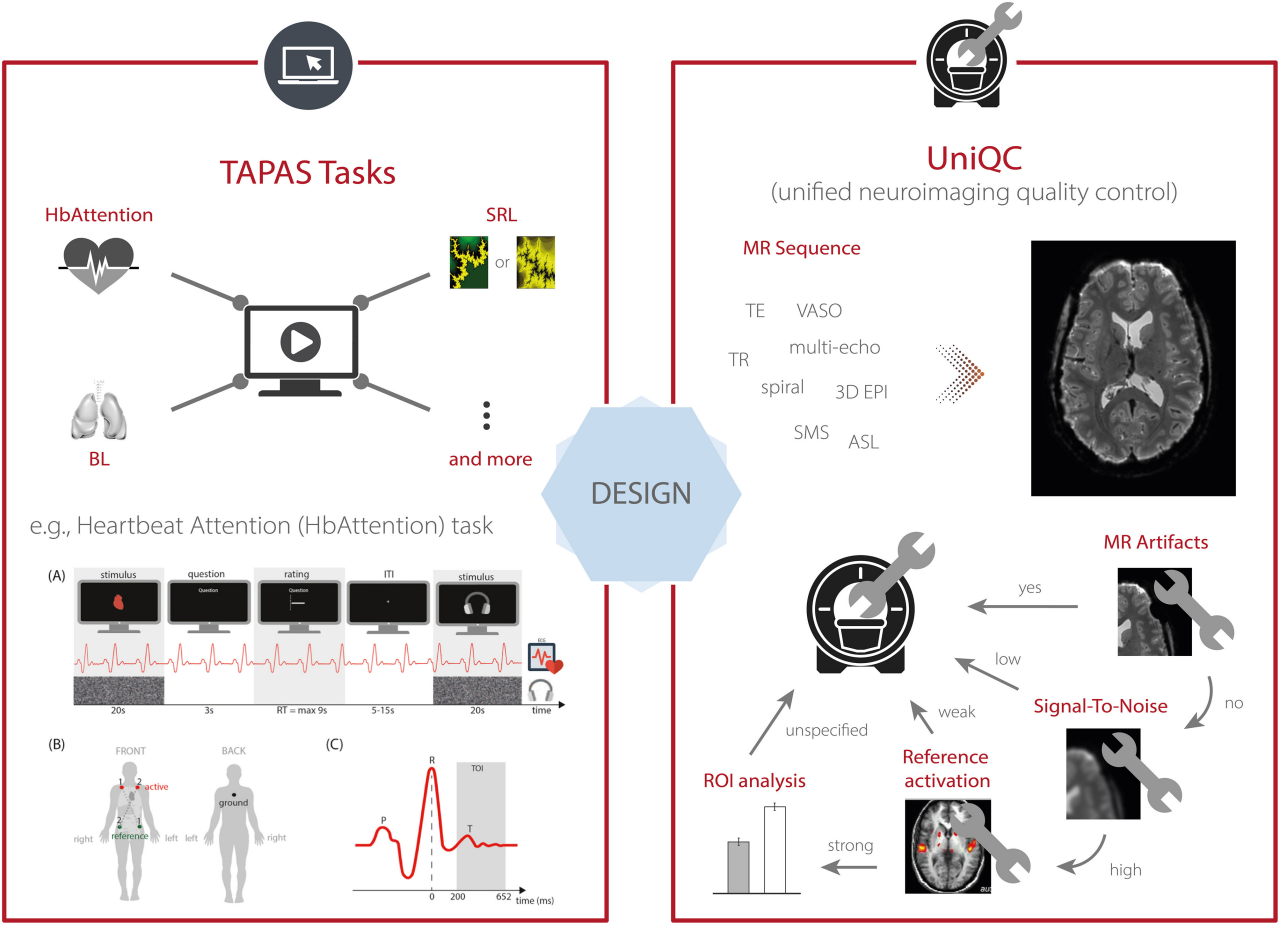
TAPAS components that aim to enhance data quality for scientific and clinical applications. This includes (*left, top*) TAPAS Tasks, a collection of experimental paradigms that have been devised and carefully tested, for instance, the Heartbeat Attention (HbAttention), stimulus-reward learning (SRL), and breathing learning (BL) task. For a complete list of tasks that are already included in TAPAS Tasks or will be included in one of the upcoming releases, see Table 1. (*Left, bottom*) Schematic overview of the experimental paradigm of the HbAttention task, as well as the placement of ECG electrodes and a typical ECG signal associated with a heartbeat [reprinted with permission from Petzschner et al. (47)]. (*Right*) Furthermore, TAPAS comprises the unified neuroimaging quality control (UniQC) toolbox which is designed to facilitate the development and optimization of MR acquisition sequences. UniQC can assess (compute and visualize) different image quality metrics (IQMs) at different stages of an fMRI experiment (i.e., from raw data to statistical images). This facilitates the acquisition of high-quality data by implementing an iterative optimization process, including basic artifact checks, temporal stability analysis, functional sensitivity analyses in the whole brain or in particular regions of interest. UniQC enables this optimization in a highly flexible fashion, independent of the exact input data (e.g., sequence, dimensionality).

To this end, TAPAS comprises the module “TAPAS Tasks” which represents a collection of experimental paradigms that have been designed and thoroughly tested (Figure 3, *top left*). TAPAS Tasks comprises several tasks that cover both the exteroceptive and interoceptive^2^ domains (for a complete list, see Table 1). For all paradigms, the stimulus code is provided as well as detailed documentation. This includes a comprehensive description of (i) the experimental task, (ii) software requirements, (iii) experimental set-up (including a list of necessary peripheral devices), and (iv) information on how to run the task.

**Table 1:** List of tasks (to be) included in TAPAS Tasks.

**Task**	**Description**
Heartbeat attention *(HbAttention)*	The HbAttention task probes differences in neural responses to heartbeats due to changes in attentional focus. The paradigm consists of alternating conditions where participants focus attention either on their heart (interoceptive condition) or on an external sound stimulus (exteroceptive condition), while keeping the sensory stimulation identical (47). The HbAttention task requires the acquisition of an ECG and can be run in the context of an EEG experiment, measuring a Heartbeat Evoked Potential (HEP), or an fMRI experiment. The task is programmed such that the stimulus timing can be easily adjusted to the experimental modality.
Heartbeat feedback *(HbFeedback)*	The HbFeedback task presents auditory-visual stimuli that are either locked to an individual's online detected heartbeat (veridical feedback about the heartbeat) or presented at a rate that is faster or slower than the individual's heartrate. The task assesses the effects of veridical vs. false feedback on physiological and neural signals related to heartbeats. It requires the simultaneous recording of EEG and ECG signals.
Heartbeat mismatch *(HbMMN)*	The HbMMN task consists of an auditory omission paradigm where a “standard” tone is presented shortly after each heartbeat, but occasionally omitted (“deviant”). In different conditions, the delay between heartbeat and tone is varied. This allows to measure changes in stimulus-evoked and heartbeat-evoked potentials between standards and omissions. In a control condition, tone presentation times are unrelated to heartbeats. EEG and ECG signals are simultaneously recorded during the task.
Filter detection *(FD)*	The FD task is a perceptual threshold breathing task where participants have to indicate on each trial whether a very small resistance (i.e., filter) or sham (i.e., empty filter) was applied to the breathing system (yes/no version) (213), or in which interval resistance was applied (two-interval forced choice version). The task is tailored to assessing respiratory interoceptive accuracy and metacognition in individual participants. Behavioral responses are recorded.
Breathing learning *(BL)*	The BL task represents an associative learning task where participants learn the association between visual cues and the subsequent presence/absence of an inspiratory resistive load. Respiratory load is applied using a novel MRI-compatible breathing system that allows for remote administration and monitoring of resistive loads and whose construction plan has been published (214). fMRI signals are recorded during the task.
Stimulus-reward learning *(SRL)*	The SRL task requires participants to predict which of two simultaneously presented visual stimuli (i.e., fractals) would yield a monetary reward. The association strengths between the visual cues and monetary outcomes change over the course of the experiment, introducing volatility. fMRI or EEG data can be acquired during the task.
Auditory mismatch negativity (aMMN)	The aMMN task is a variant of the auditory oddball paradigm in which the degree of volatility in the auditory stream varies over time. While engaging in a visual distraction task, participants passively listen to repeated presentations of a high and a low tone. During stable phases of the experiment, one stimulus reliably serves as the “standard” (more frequent) tone and the other one as the “deviant” tone. During volatile phases, the roles of standard and deviant switch more rapidly. Deviance processing can be compared between different levels of stability/volatility. Task versions are available for both EEG or fMRI recordings.
Visual mismatch negativity (vMMN)	The vMMN task implements the identical probabilistic stimulus sequence as the aMMN task. However, instead of auditory stimuli, Gabor patches of different orientations are used to probe mismatch responses in the visual domain. Task versions are available for both EEG and fMRI recordings.
Antisaccades (AS)	The AS task asks participants to perform antisaccades which are a type of voluntarily controlled eye movements. In TAPAS, code is available to run two different versions of the AS task (215, 216) using an EyeLink (SR Research, Ottawa, ON, Canada) eye tracking system. The versions of the task differ in the timing (with or without a delay before the eye movement) and position (at center or at peripheral target position) of the presentation of the task cue.

*TAPAS Tasks will include a variety of different paradigms that probe exteroceptive and/or interoceptive processes. So far, the Filter detection (FD) and Breathing learning (BL) tasks are included; the other tasks will follow as soon as the respective papers are published*.

Here, as an example, we describe the *Heartbeat Attention (HbAttention)* task in more detail (Figure 3, *bottom left*). The task implements a novel paradigm to probe purely attentional differences of the heartbeat evoked potential between exteroceptive and interoceptive conditions (47). The paradigm consists of alternating conditions where participants are asked to focus attention either on their heart or on a simultaneously presented auditory stimulus (white noise). Importantly, in both conditions the sensory stimulation is identical. Using this paradigm, Petzschner et al. (47) found an increased heartbeat evoked potential during interoceptive compared with exteroceptive attention. A non-invasive readout of the attentional modulation of interoceptive processes could potentially be of high clinical relevance, since alterations in interoceptive processing have been recognized as a major component of various psychiatric conditions, including mood and anxiety disorders, eating disorders, drug addiction, as well as depression (48).

TAPAS Tasks represents work in progress and not all experimental paradigms listed above are available yet (for more details, see Table 1). Newly devised experimental paradigms will be added in the future. We hope that by making these tasks openly available to the community, TAPAS Tasks may contribute to growing a collection of standardized experimental paradigms in TN/CP.

### Optimization of MRI Protocols

In the context of neuroimaging, data quality also depends heavily on the MR scanner settings and acquisition sequence. Carefully crafted sequences with optimized parameter choices can offer considerable gains in functional sensitivity and specificity for the targeted research question (49–51). However, due to the large variety of available parameters and their interdependency, optimization of MR protocols is challenging and suboptimal acquisition choices might reduce data quality, e.g., low signal-to-noise ratio or pronounced artifacts like ghosting, ringing, signal dropouts and distortions due to magnetic field inhomogeneities [for an overview, see (52–55)]. Hence, the development, optimization and validation of robust and powerful MR protocols prior to data acquisition is critical and tools are needed that ease this process.

To this end, TAPAS includes the *unified neuroimaging quality control* (UniQC) toolbox (56), which provides a framework for flexible, interactive and user-friendly computation and visualization of various quality measures in neuroimaging data (Figure 3, *right*). UniQC facilitates fast prototyping and optimization of acquisition sequences by providing tools for artifact detection and sensitivity analyses across the entire image or tailored toward specific regions of interest.

As sequence development constitutes an iterative process, feedback on image quality has to be immediate and specific to the protocol changes, so that the performed quality control (QC) query informs the operator on how to adjust parameters for the next scan (Figure 3, *right*). For example, if an unexpected bias field occurs in the mean image, both excitation and receiver channels could be compromised. In this case, fast display of individual coil images is critical, which would be omitted if the mean image were inconspicuous. Thus, QC during sequence development resembles a decision tree, where the outcome of one image quality metric (IQM) determines the selection of the next one, with varying display options. This necessitates an interactive, fast and flexible way to compute and visualize IQMs. Typically, such a decision tree starts from basic artifact checks over temporal stability all the way to functional sensitivity analyses in particular regions of interest, with the occasional return to the scanner, if a QC step fails (Figure 3, *right*).

To achieve this functionality, UniQC exploits the object-oriented programming capabilities in MATLAB. Importantly, UniQC is not restricted to 4-dimensional neuroimaging data (i.e., space and time) like most other software packages, but generalizes operations to n-dimensional data. This generalization to arbitrary numbers of dimensions comes in useful when handling data from multiple receiver coils (57), as well as multi-echo (58, 59) or combined magnitude/phase fMRI data (60–62) in a single unified framework. Similarly, prominent non-BOLD fMRI contrasts rely on an additional tag/control dimension, for instance, Vascular Space Occupancy [VASO; (63, 64)] or Arterial Spin Labeling [ASL; (65, 66)], with the former being particularly relevant for depth-dependent fMRI. Furthermore, UniQC allows seamless integration with SPM and other MATLAB toolboxes in order to benefit from image and fMRI processing algorithms that are already available in those packages.

In summary, UniQC provides a flexible, interactive and user-friendly toolbox for evaluating MR pulse sequence development and quality control of n-dimensional neuroimaging data. These efforts carry over from the design stage to the data collection, in that UniQC allows utilizing the processing and visualization pipeline established here directly as a quality control protocol—enabling unique QC toward the aims of each study.

## Conduct, Check and Correct

Besides optimization steps prior to data acquisition, further steps are necessary during and after the measurement to ensure adequate data quality. Specifically, ongoing monitoring of data quality during image acquisition is critical, because both the MR system and the study participant constitute significant potential noise sources. On the system side, data quality across different time points and scanning sites may vary due to potential malfunctions or alterations in the scanner hardware, that must be detected in a timely manner. On the participant's side, even under ideal circumstances, with tailored experimental designs, optimized acquisition sequences and thorough quality control measures, fMRI data is still subject to artifacts outside the experimenter's control [e.g., motion, physiology; (67, 68)]. Adequately correcting for these artifacts is essential to avoid bias in subsequent data analyses and to ensure that conclusions are not confounded [e.g., (69–71)]. In what follows, we elaborate on these points and describe tools in TAPAS that address these challenges.

### Quality Monitoring

fMRI analyses rely on the content of brain (or spinal cord) images as information source. Thus, the visual inspection of raw images by one or multiple experts is still often considered a gold standard for quality control. However, visual assessment depends on individual rater experience and visualization choices (e.g., slice orientation, windowing) which may reduce the apparent information content of an image to detect artifacts or improper acquisition parameters (72) and generally aggravates inter-rater reliability. Furthermore, limited time resources and fatigue make the naïve visual inspection of every raw image quickly unfeasible, as even a single fMRI dataset contains hundreds of volumes with dozens of slices each. This challenge is exacerbated for large-scale datasets, like the Human Connectome Project [HCP; (46)] or UK Biobank (73), where thousands of participants are measured.

Automation of quality control is therefore required, and can in principle target both aspects of the manual image classification by raters. A first approach is to replace raters by a machine learning algorithm working on derived IQMs to reduce the high-dimensional feature space of image time series. A second option is that the expertise of the rater can be harnessed more efficiently by providing flexible tools for image manipulation and visualization with intuitive interfaces to derive and inspect relevant IQMs, thereby reducing operator fatigue and inconsistency.

The first approach was explored early on for anatomical T1-weighted images (74) and demonstrated good discriminability of undistorted, noisy, and distorted images based on a subset of 239 IQMs. Since then, various additional tools for automatic quality control have been proposed, introducing additional IQMs to assess image quality (75–77). These methods have been refined and extended into scalable QC frameworks for large-scale fMRI studies, most notably in the form of the MRI Quality Control tool [MRIQC; (78)] and within the UK biobank study (79). Their key advancement lies in the ability to classify images in a binary fashion (“good” vs. “problematic”) or even categorizing multiple artifact classes (79). Standardized quality reports then provide guidance, as to whether data from a given participant should be included in subsequent analyses or not. In order to ensure accurate classification, these algorithms are typically trained on large curated datasets that include both patients and healthy controls [e.g., ABIDE (80) and DS030 (81) for MRIQC].

While such fully automatized approaches with minimal visual output and manual assessment might be the only viable solution in studies with thousands of participants, the required high degree of standardization of the acquisition protocol as well as the need for large, representative training datasets poses limitations on its utility. In TN/CP, less well-studied clinical populations and novel technologies can pose challenges when trying to exploit established mappings between IQMs and image quality. In particular, this can occur when employing advanced imaging hardware or acquisition sequences to maximize sensitivity for individual subject measurements, IQMs may fall outside the standard range or become inapplicable. For example, higher magnetic field strengths and customized high-density or surface coils (82, 83) induce atypical image intensity variations (bias fields). Similarly, Nyquist ghosts manifest differently for spiral readouts than in conventional Cartesian echo-planar imaging. Thus, fMRI data may require different IQMs or thresholds when deviating from standard acquisition choices.

**Figure 4:**
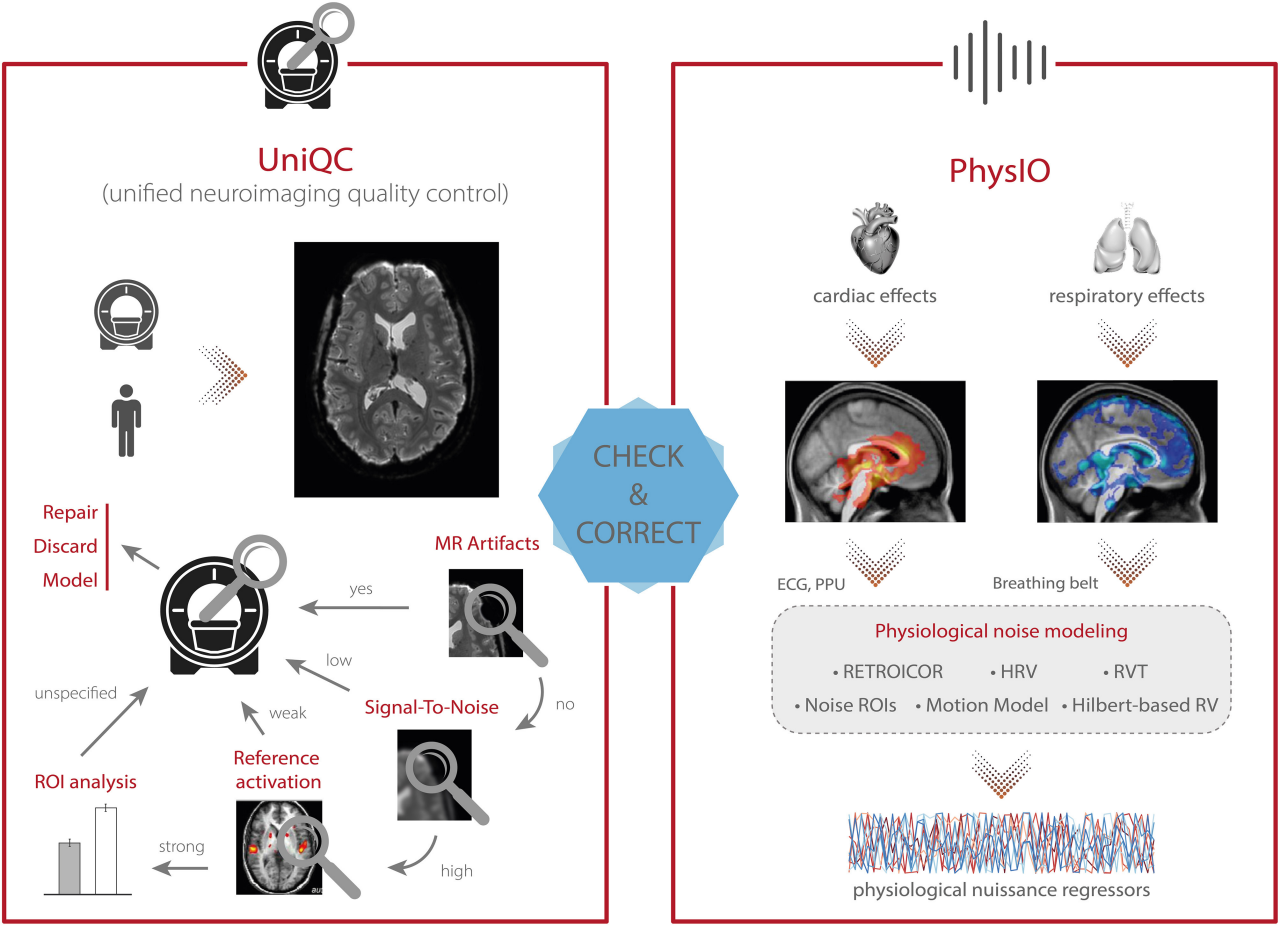
TAPAS components that aim to monitor data quality and correct for physiological confounds. **(Left)** In addition to supporting the development and optimization of MR acquisition sequences (see Figure 3), UniQC also facilitates monitoring of data quality during data acquisition in order to quickly identify potential problems in the acquisition processes which might relate to malfunctions or alterations in the scanner hardware, as well as to artifacts related to the participant (e.g., motion, physiology). **(Right)** PhysIO implements model-based physiological noise correction based on peripheral recordings of cardiac [e.g., electrocardiogram (ECG), photoplethysmographic unit (PPU)] and respiratory (e.g., breathing belt) cycle. The toolbox uses RETROICOR as well as modeling of the impact of heart rate variability (HRV) and respiratory volume per time (RVT) on the BOLD signal (e.g., using Hilbert-based respiratory volume) to derive physiological nuisance regressors that can be utilized in subsequent statistical analyses to account for physiological confounds in fMRI signals.

In these domains, the second option for QC automation is preferable. This approach empowers the rater to determine which IQMs to inspect, how to visualize them, and at which stage of the analysis stream to assess them. While parts of this approach have been implemented by providing standardized visual reports (e.g., MRIQC, fMRWhy) or interactive QC visualization tools (e.g., visualQC), a comprehensive framework that integrates all these functionalities has been missing. To address this, we designed the UniQC toolbox to meet these demands by offering flexible, interactive and user-friendly assessment of fMRI data (Figure 4, *left*). Importantly, the quality control pipeline derived during the sequence design stage (section Design) can be readily deployed to cover QC automation, and once quality issues are detected, UniQC also provides a framework to “interrogate” the data efficiently and identify potential causes of the problem.

In principle, this fast and precise identification can lead to quality improvements in three ways (Figure 4, *left*). First, the information can be used to isolate and repair hardware malfunction (e.g., of certain coil elements) to swiftly restore quality levels for the next scan or participant. Second, the quality of the affected dataset can be increased by modeling the impact of distinct noise sources, as identified by the QC decision tree [e.g., electrostatic spike artifacts in the images or interactions between subject motion and magnetic field; (84–86)]. Third, selectively discarding the low-quality data only, as isolated by the customized QC interrogation, salvages quality levels for the remainder of the data (Figure 4, *left*).

Furthermore, unlike generic QC pipelines, the customization afforded by UniQC facilitates testing whether any given dataset shows a functional response relevant for the research question. For example, due to the seamless integration with other MATLAB toolboxes such as SPM, UniQC can analyze statistical maps from study-specific GLMs, as well as provide region-of-interest (ROI) statistics. With this functionality, task-fMRI performance can be validated using robust expected activation patterns as a sanity check, for example, sensory-motor activation during a learning task, before proceeding to more complex analyses. Thus, UniQC offers flexible, interactive and study-specific quality control of the image acquisition system and the imaged participant. Thanks to its modularity, UniQC can be further integrated to monitor quality throughout the preprocessing stage (section Preprocessing), independent of the concrete pipeline.

### Physiological Noise Modeling

One of the main confounds in fMRI is physiological noise as it perturbs blood oxygen level dependent (BOLD) signals (68, 87)—which can substantially hamper both classical fMRI analysis as well as computational modeling of the data. The two primary sources of physiological noise are the cardiac and respiratory cycles (88). The respiratory cycle introduces confounds by distortions of the magnetic field due to the movement of the participant's chest (89) as well as bulk susceptibility variation in the lungs (90). Additionally, the respiratory cycle alters the pressure of blood CO_2_ (which is a vasodilator) over longer time periods, thereby inducing slow signal fluctuations (91). The cardiac cycle, on the other hand, modulates blood volume and vessel diameter during systole and diastole, leading to small deformations of brain tissue and brainstem displacement, causing periodic motion of the cerebrospinal fluid (92). Furthermore, variability in heart rate induces alterations in the oxygen level in the blood (93), and consequent low frequency signal fluctuations (94). Finally, interactions between the cardiac and respiratory cycles, such as in respiratory sinus arrhythmia (i.e., accelerated heartbeat during inhalation), induce additional non-trivial physiological fluctuations (95).

Various physiological noise correction methods for fMRI exist, either based solely on the fMRI time series and prior assumptions of spatiotemporal noise properties, or modeling the noise from independent physiological recordings (e.g., using electrocardiogram (ECG), photoplethysmographic unit (PPU), and breathing belts) (88, 96). Arguably, for TN/CP applications with clinical populations and pharmacological interventions, methods based on independent recordings might be preferable: In clinical populations, physiological processes that impact on the BOLD signal may differ from priors that were defined based on the general population.

Several freely available implementations for model-based physiological noise correction are available, including AFNI 3DRETROICOR (67), FSL Physiological Noise Modeling (97), and PhLEM (98); however, relatively few studies have capitalized on these tools, in particular for task-based fMRI. One important practical challenge—that is exacerbated in clinical populations with less compliant subjects—is the variable data quality of the peripheral recordings which these models are based on. Reduced data quality may be due to subject motion or (partial) detachment/saturation of the peripheral devices. In most implementations, preprocessing of these recordings is minimal, and identification of the physiological cycles typically relies on peak detection provided by the MR scanner vendor (96). Alternatively, manual intervention to correct erroneous detection is offered, hampering the development of automatized pipelines and the translation of physiological noise modeling into routine application.

The *PhysIO* toolbox (96) in TAPAS offers methods for model-based physiological noise correction based on peripheral recordings of the cardiac (e.g., ECG, PPU) and respiratory cycles (e.g., breathing belt). PhysIO utilizes these peripheral measures to model the periodic effects of pulsatile motion and field fluctuations using RETROICOR (67). Furthermore, the toolbox accounts for end-tidal CO_2_ changes and heart rate-dependent blood oxygenation by convolving respiratory volume per time (RVT) and heart rate variability (HRV) with a respiratory and cardiac response function, respectively (91, 99, 218). For these methods, emphasis is placed on robust preprocessing of the input time series via reliable peak detection in low-SNR regimes, as well as a novel method for RVT estimation using the Hilbert transform (100). As a more data-driven alternative for noise correction, PhysIO also allows the extraction of signals from pre-defined regions of interest (“noise ROIs”) as additional confound regressors—for instance, signal related to white matter or cerebrospinal fluid (CSF). Finally, the toolbox also incorporates various strategies for correcting motion-related artifacts by implementing, for instance, the Volterra expansion confound set (101) or censoring strategies based on the framewise displacement (69). A schematic illustration of the modeling process in PhysIO is provided in Figure 4, *right*. PhysIO provides both command-line operation for de-noising multiple subjects conveniently, as well as a user-friendly graphical interface within the SPM Batch Editor. Thereby, physiological noise correction can be integrated with complete fMRI preprocessing pipelines, minimizing the need for manual interventions or custom programming (96). Additionally, PhysIO ensures robust preprocessing even for low-quality data and provides simple diagnostic tools to assess the correction efficacy in individual subjects. This renders PhysIO an accessible noise correction tool for preprocessing pipelines both in basic neuroscience studies as well as for clinical purposes.

## Preprocessing

Data preprocessing is closely intertwined with quality control and artifact correction. Thorough preprocessing is particularly important for complex data acquired using neuroimaging techniques such as fMRI or EEG/MEG (102). To this purpose, researchers typically create *ad hoc* preprocessing workflows for each study individually (103), building upon a large inventory of available tools. Broadly speaking, preprocessing steps can be separated into two main categories: (i) preprocessed time series are derived from the original data after application of retrospective signal corrections, spatiotemporal filtering, and resampling in a target space (e.g., MNI standard space), and (ii) confound-related information in the data can be modeled or taken into account through nuisance regressors (i.e., regressors of no interest) in subsequent statistical analyses using the general linear model (GLM). These confounds may include motion parameters, framewise displacement, physiological (cardiac or respiratory) signals, or global signals (88, 104).

These and additional procedures for preprocessing neuroimaging data are available in various software packages including, SPM (32), FSL (105), FreeSurfer (106), AFNI (34), or Nilearn (107). The plethora of different preprocessing tools and workflows manifests in the absence of a current gold standard for preprocessing neuroimaging data, despite several attempts to establish best-practice guidelines (102, 108–110).

In an attempt toward common preprocessing standards, large-scale consortia like the HCP or the UK Biobank provide access not only to the raw data, but also to already preprocessed versions of the data. For instance, in the HCP database, researchers have access to the version of the data which have been subjected to a common minimal preprocessing pipeline (111). However, these workflows are usually tailored toward the particular dataset's idiosyncrasies and do not readily translate to other datasets. A first attempt toward such a universally applicable preprocessing workflow is fMRIPrep (112), which represents a pipeline that combines tools from several of the above-mentioned software packages. fMRIPrep autonomously adapts the workflow to the present data, rendering the approach robust to data idiosyncrasies and potentially applicable to any dataset without manual intervention.

While no toolbox dedicated to data preprocessing is currently available in TAPAS, our tools from the previous step (i.e., “Conduct, Check and Correct”) integrate well with most of the third-party software packages highlighted above. For instance, PhysIO integrates seamlessly with the batch editor system of SPM to facilitate the derivation of nuisance regressors related to physiological confounds that can be utilized in GLM analyses. Similarly, UniQC is designed to integrate with SPM and other MATLAB toolboxes. Importantly, PhysIO and UniQC are also designed to integrate well with other neuroimaging software packages. For instance, PhysIO stores all physiological noise regressors in a dedicated text file which can be inputted into the first-level analyses in any software package (e.g., FSL, AFNI).

## Inference

Once neuroimaging and/or behavioral data have been preprocessed and artifacts have been corrected, the question arises how best to interrogate the data in order to gain insights into the functioning of the human brain and alterations thereof in disease. Concerning clinically oriented studies, it has been pointed out (4, 25) that focusing on differences in descriptive measures—such as BOLD activation, functional connectivity patterns or task performance—between patients and healthy controls is unlikely to result in improvements of clinical practice. This is because these analyses do not provide an understanding of the symptom-producing mechanisms and they do not easily inform the development of biologically grounded clinical tests. Consequently, these measures have not yet led to routine applications in clinical practice (4, 113).

To address this shortcoming, mathematical models of neuroimaging and behavioral data that capture putative physiological and cognitive disease mechanisms may represent a promising avenue. This line of thinking is at the core of clinically-oriented modeling disciplines like Computational Psychiatry (15, 16, 22–24, 27), Computational Neurology (15, 114), and Computational Psychosomatics (115). For example, in Computational Psychiatry, a major goal is to move from the current syndromatic nosology to disease classifications based on computational assays that may improve differential diagnosis and treatment prediction for individual patients (7, 116).

TAPAS contributes to this endeavor by providing a collection of computational tools that can be applied to neuroimaging (fMRI) or behavioral data (decisions, eye movements). All of these approaches are so-called generative models (117). Generative models specify the joint probability *p*(*y*, θ|*m*) over measured data *y* and model parameters θ, which—according to probability theory—can be written as the product of the likelihood function *p*(*y*|θ, *m*), representing the probability of the data given a set of model parameters, and the prior distribution *p*(θ|*m*), encoding the *a priori* plausible range of parameter values. Together, likelihood and prior yield a probabilistic forward mapping from latent (hidden) states of a system (e.g., neuronal dynamics) to observable measurements (e.g., BOLD signal). Model inversion enables inference on the parameters and latent states of the system from measured data, and can be accomplished using a variety of approximate Bayesian techniques (e.g., variational Bayes, Markov chain Monte Carlo or Gaussian process optimization). Their ability to reveal latent mechanisms underneath the visible data and their natural connection to hypothesis testing through model selection procedures (118) have established generative modeling as a cornerstone of TN/CP (13, 119).

In brief, the generative models included in TAPAS comprise: (i) models of effective (directed) connectivity among neuronal populations, (ii) models of perception in the light of an uncertain and volatile environment, as well as (iii) models of inhibitory control. In what follows, we briefly describe the different generative models in TAPAS and highlight how each of them might be useful for clinical (neuro)modeling.

### Generative Models of Neuroimaging Data

Models of effective connectivity describe the mechanisms by which neuronal populations interact and how these mechanisms give rise to measured data (e.g., fMRI or EEG/MEG). By inverting these generative models, it is possible, in principle, to infer on the directed (synaptic) influences neuronal population exert on one another (120, 121). This differs from measures of functional connectivity (e.g., Pearson's correlation) which are essentially descriptive and undirected statistical indices. Models of effective connectivity hold particular promise for TN/CP, since global dysconnectivity has been proposed as a hallmark of various mental disorders (10, 122), including schizophrenia (31, 123–125, 164), autism (126–128), and depression (129–131).

A frequently used generative modeling framework for inferring effective connectivity from neuroimaging data is dynamic causal modeling [DCM; (132)]. In brief, DCM describes changes in neuronal activity as a function of the directed interactions among neuronal populations and experimental manipulations that can perturb the system. DCM was initially introduced for fMRI (132) and later extended to electrophysiological data (133). Comprehensive reviews on DCM can be found elsewhere [e.g., (121, 134–136)]. DCM is freely available as part of SPM and has found widespread application. For example, with regard to clinical applications, DCM has been used to study schizophrenia (137–141), autism (142, 143), and depression (144, 145). Despite these promises, the classical DCM approach is also subject to several limitations—which may become particularly relevant in the context of TN/CP, where the goal is to develop computational assays that inform prediction of clinical trajectories and treatment responses in individual patients. In what follows, we highlight some of these limitations and outline how the methodological advances in DCM included in TAPAS aim to address these challenges.

#### Global Optimization

When translating computational advances like DCM into computational assays, the robustness of the inference procedure and the reliability of the parameter estimates become paramount (146). Standard model inversion in DCM rests on variational Bayes under the Laplace approximation [VBL; (147)] which is computationally efficient, yet subject to several limitations (134): First, VBL rests on maximizing the negative free energy (which serves as a lower bound approximation to the log model evidence) using gradient ascent and is thus inherently susceptible to local maxima if the objective function is multimodal. Second, even when the global maximum is found, the distributional assumptions (i.e., Laplace and mean-field approximations) might not be justified, potentially rendering the approximate posterior distribution a poor representation of the true posterior. Third, when the distributional assumptions of the Laplace approximation are violated, the negative free energy is no longer guaranteed to represent a lower bound on the log model evidence (148).

Sampling-based model inversion schemes, typically based on Markov chain Monte Carlo (MCMC) methods, do not require any distributional assumptions about the posterior and are guaranteed to be asymptotically exact (i.e., converge to the global extremum in the limit of infinite samples). This renders sampling-based methods an appealing alternative to VBL. However, they come at the cost of other challenges: First, sampling-based routines are computationally expensive. Second, convergence is only guaranteed in the limit of infinite samples; detecting convergence in practice thus rests on heuristics. Third, unlike VBL, sampling-based methods do not readily provide an estimate of the (log) model evidence, but require additional strategies, which further aggravate the computational burden. For instance, the current gold standard for sampling-based estimates of the model evidence, thermodynamic integration [TI; (149–151)], requires running multiple MCMC chains at different “temperatures” (i.e., at different positions along a path from prior to posterior). Until recently, these reasons have been prohibitive for the use of sampling-based model inversion for DCMs.

**Figure 5:**
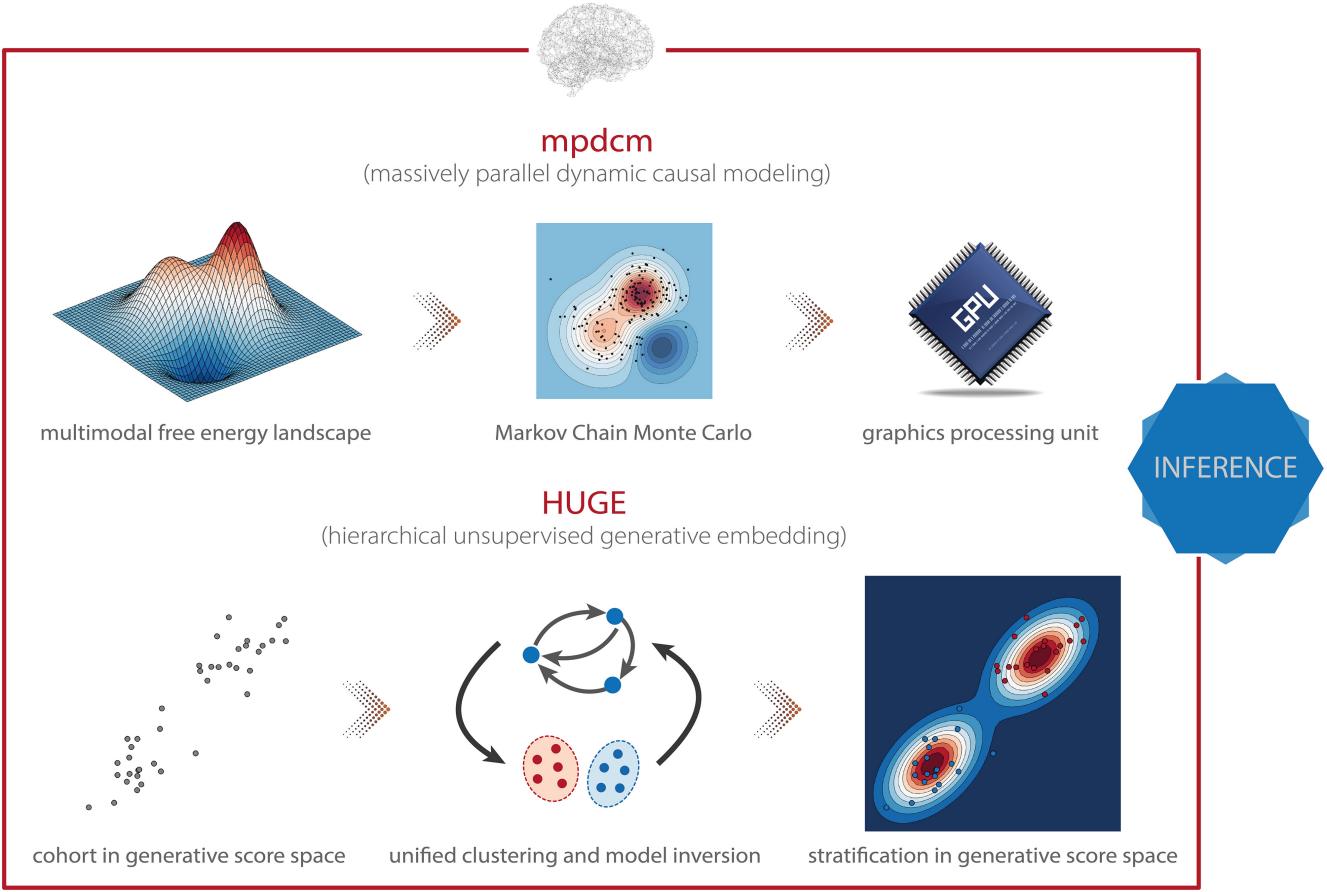
TAPAS components that implement generative models of neuroimaging data (I). **(Top)** Massively parallel dynamic causal modeling (mpdcm) renders sampling-based model inversion computationally feasible by exploiting graphics processing units (GPUs). This allows one to obtain more faithful results in the presence of a multimodal optimization landscape. **(Bottom)** Hierarchical unsupervised generative embedding (HUGE) combines the inversion of single-subject DCMs and the clustering of participants into mechanistically homogenous subgroups within a single generative model.

The *massively parallel dynamic causal modeling* (mpdcm) toolbox (152) implemented in TAPAS renders sampling-based model inversion in the context of DCM for fMRI computationally feasible (Figure 5, *top*). This is achieved by exploiting the power of graphics processing units (GPUs) for the evaluation of the likelihood function, which represents the computationally most expensive operation, as it requires integration of differential equations in the neuronal and hemodynamic models. Importantly, mpdcm even makes the evaluation of the model evidence via thermodynamic integration computationally feasible. In a recent preprint, Aponte et al. (217) demonstrated that TI provides more accurate and robust estimates of the model evidence than VBL, while computational demands are kept at a moderate level.

Beyond the mpdcm toolbox, which is designed to support DCM for fMRI, other gradient-free and gradient-based MCMC sampling schemes have also been introduced to DCM for electrophysiological data (153, 229). However, these tools have not yet been made publicly available.

#### Empirical Bayes for DCM

Another challenge concerns the specification of prior distributions in DCM, which have been found to profoundly impact the posterior estimates and their reliability (154). Notably, in the context of hierarchical Bayesian models, there is a principled way of estimating priors by exploiting measurements from multiple subjects: empirical Bayes [EB; (155–157)]. In brief, in EB, the posterior density at any given level is constrained by the level above. For instance, in a two-level hierarchical model, observed data *y* = {*y*_1_, *y*_2_, …, *y*_*n*_} are assumed to be generated from a set of latent (hidden) parameters θ = {θ_1_, θ_2_, …, θ_*n*_} according to the likelihood *p*(*y*|θ, *m*). In turn, the parameters θ are considered to represent samples from a population density *p*(θ|η, *m*), where η refers to the hyperparameters (117, 158). Consequently, under the hierarchical structure of a multi-subject or mixed-effects model, inference on the single-subject level is constrained by the group-level information. These constraints are then referred to as empirical priors since they are informed by the empirical data (of the entire group). A special case of EB is referred to as parametric empirical Bayes (PEB), where the hyperparameters η are approximated using the maximum likelihood estimate or a moment expansion, which allows one to express the hyperparameters in terms of the empirical mean and variance (159). One particular variant of PEB is the Gaussian-Gaussian model, where single-subject data are assumed to be generated by adding Gaussian noise to the group mean (160).

The *hierarchical unsupervised generative embedding* (HUGE) toolbox contained in TAPAS implements EB in the context of DCM for fMRI (161, 162). HUGE combines the inversion of single-subject DCMs and the clustering of subjects into mechanistically homogenous subgroups into a single generative model (Figure 5, *bottom*). This is achieved by combining the non-linear DCMs at the individual level with a mixture-of-Gaussians clustering model at the hierarchically higher level (117, 158). In other words, HUGE assumes that each individual from a population of *N* subjects belongs to one of at most *K* subgroups or clusters. The DCM parameters θ for all subjects from one cluster *k* are then assumed to be normally distributed with distinct mean μ_*k*_ and covariance matrix Σ_*k*_. This cluster-specific normal distribution effectively means that different prior distributions apply over subjects, depending on which subgroup they belong to, and that these priors are learned from the data (i.e., subgroup-specific EB). Hence, in principle, the framework is capable of stratifying heterogeneous spectrum disorders, as defined by DSM/ICD, into subgroups that share common pathophysiological mechanisms (for more details, see below). Importantly, HUGE also implements “pure” EB by fixing the number of clusters to one and merely exploiting the hierarchical dependencies in the data. This effectively switches off the clustering model. The utility of this mode of operation has been demonstrated in simulations by Yao et al. (162), highlighting the expected shrinkage effect (reduced variability) of the posterior parameter estimates toward the population mean observed in EB (117). Parameter estimation in HUGE can be performed by employing either a sampling-based MCMC inversion scheme (161, 163), which is asymptotically exact yet (relatively) slow, or a VB implementation (162), which is computationally more efficient yet might be vulnerable to local extrema. Notably, at the moment, only the VB implementation of HUGE is available in TAPAS. The sampling-based variant will be published as part of an upcoming release of the toolbox.

#### Whole-Brain Effective Connectivity Analysis

Apart from the computational and statistical challenges mentioned above, a conceptual concern is that DCMs are typically restricted to relatively small networks in order to keep model inversion computationally feasible. While this may be advantageous in some cases by enforcing a theory-driven analysis of high-dimensional and noisy fMRI data, it can also represent a limiting factor. Specifically, many cognitive processes, as well as the “resting state” (i.e., unconstrained cognition in the absence of experimental manipulations), engage a widespread network that cannot be captured faithfully by a handful of nodes. Furthermore, in the context of Computational Psychiatry, putative pathophysiological processes underlying various mental disorders have been linked to global (large-scale) alterations of functional integration in brain networks [e.g., (31, 123, 125–127, 129, 130, 164)]. This calls for the development of computational models that are capable of inferring effective (directed) connectivity in whole-brain networks (122).

**Figure 6:**
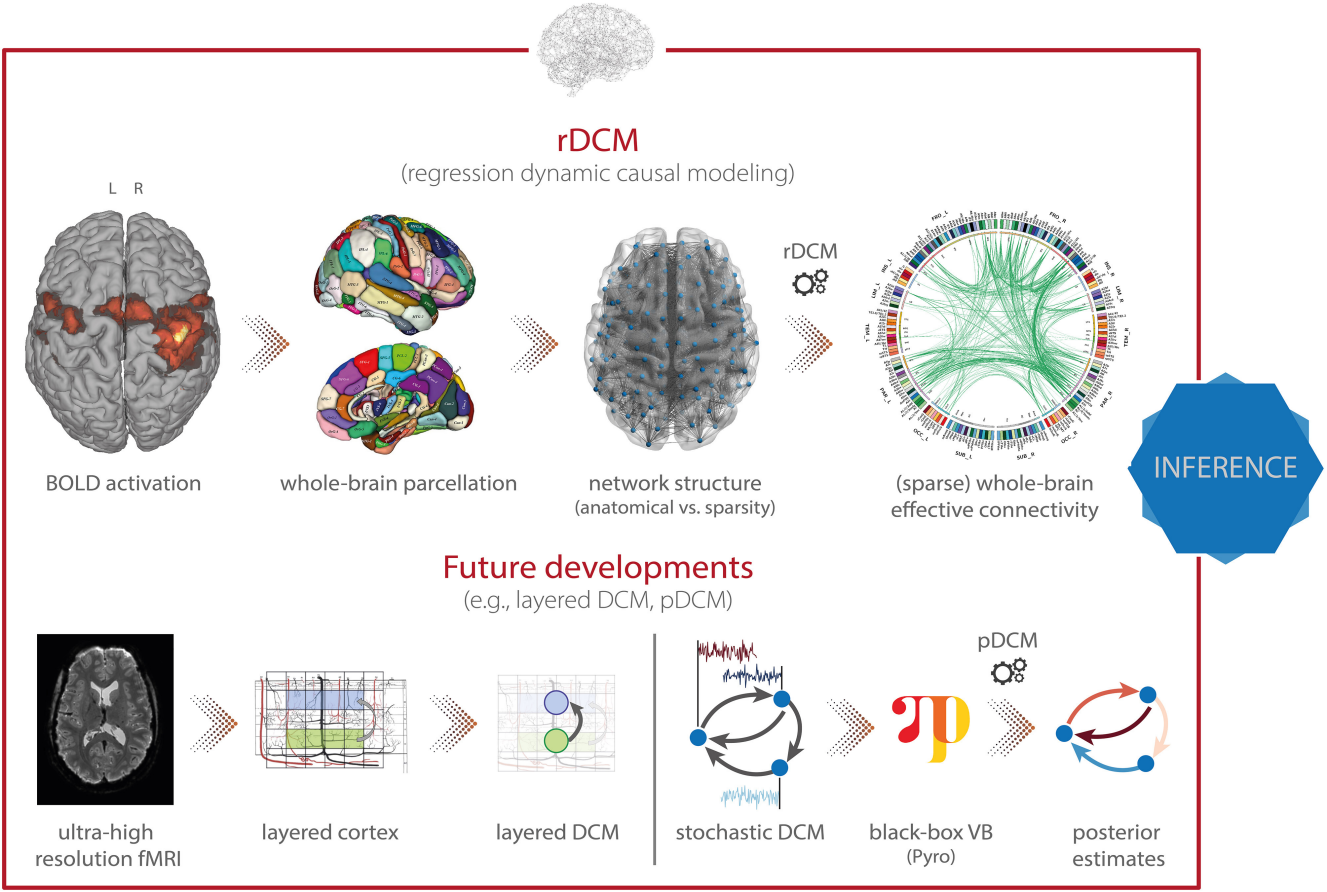
TAPAS components that implement generative models of neuroimaging data (II). **(Top)** Regression dynamic causal modeling (rDCM) is a novel variant of DCM for fMRI that scales gracefully with the number of nodes and thus makes whole-brain effective connectivity analyses feasible. **(Bottom)** Layered DCM (l-DCM) and pDCM as future developments of DCM, representing tools that will be included in TAPAS in one of the next upcoming releases. Parts of figure reproduced with permission from Heinzle et al. (169), Copyright 2016 Elsevier, and (167), Copyright 2020 Elsevier.

*Regression dynamic causal modeling* [rDCM; (165, 166)] represents a recent variant of DCM that renders model inversion extremely efficient. This is achieved by converting the numerically costly estimation of coupling parameters in differential equations of a linear DCM in the time domain into a Bayesian linear regression model in the frequency domain (Figure 6, *top*). Under a suitably chosen mean-field approximation, analytically solvable VB update equations can be derived for this model. The ensuing computational efficiency allows rDCM to scale gracefully to large-scale networks that comprise hundreds of regions. Furthermore, rDCM has recently been augmented with sparsity constraints to automatically prune fully connected networks to an optimal (in terms of maximal model evidence) degree of sparsity (165). This is achieved by introducing binary indicator variables into the likelihood function, which essentially serve as feature selectors. For this generative model, comprehensive simulation studies demonstrated the face validity of rDCM with regard to model parameter and model architecture recovery. Furthermore, we have provided initial demonstrations of the construct validity of the approach in applications to empirical data. For instance, using ultra-high field (7T) fMRI data from a simple hand movement paradigm with the known relevant connections, we demonstrated that rDCM inferred plausible effective connectivity patterns in whole-brain networks with more than 200 regions (167). Furthermore, we have recently demonstrated that rDCM can not only be applied to task-based, but also to resting-state fMRI data (168). Notably, inversion of whole-brain models with rDCM is computationally highly efficient on standard hardware: even for whole-brain networks with more than 200 regions, it takes only a couple of minutes for fixed network architectures, and a few hours when pruning fully connected networks.

#### Future Developments

Besides the toolboxes mentioned above, which are already part of TAPAS, additional variants of DCM for fMRI will be released soon. Specifically, this includes: (i) layered dynamic causal modeling [layered DCM; (169)], and (ii) pDCM, a Python-based DCM implementation focused on amortized inference of stochastic DCMs using novel probabilistic-programming techniques. Here, we briefly outline these two advances.

First, layered DCM addresses challenges in effective connectivity analyses that become relevant when moving toward high-resolution fMRI measurements at the sub-millimeter scale (Figure 6, *bottom left*). This allows differentiating BOLD signals from different cortical layers, an important aspect for testing desiderata of modern theories of brain structure and function. Specifically, prominent “Bayesian brain” theories like predictive coding (170, 171) postulate that supragranular and infragranular cortical layers convey different signals via their efferent cortico-cortical connections. Testing these theories might not only further our understanding of the functioning of the human brain in health, but also has important implications for delineating pathophysiological processes in disease.

However, for layered fMRI data, the spatial layout of cortical blood supply—in particular, the venous blood draining back from lower layers to the cortical surface—confounds responses in different layers and thus renders interpretations non-trivial. Accounting for such draining effects is thus important, yet not readily possible within contemporary hemodynamic models, such as the Balloon model currently implemented in DCM (172–174) or more recent hemodynamic models that strive for increased biological plausibility (175). Layered DCM addresses this limitation by extending the classical Balloon model with a phenomenological description of blood draining effects. This rests on including a delayed coupling of the relative blood volume and deoxyhemoglobin concentration across layers. For this framework, Heinzle et al. (169) demonstrated the face validity using simulation studies, as well as the practical utility in an application to empirical fMRI data from a simple visual paradigm. While a detailed dynamic model of layered blood flow effects was published recently (176), this model requires more parameters and full model inversion was not explored in this paper.

Second, a novel inversion scheme for stochastic DCMs will be included in TAPAS, leveraging recent breakthroughs in black-box variational inference. In brief, the approach makes use of optimization algorithms from deep learning software packages to allow inference of general probabilistic models (Figure 6, *bottom right*). For inference, one directly infers the neuronal and hemodynamic parameters, but temporal convolutional neuronal networks are used to amortize the inference of the neuronal states themselves. This allows inferring the hidden (neuronal) states for any length of input data while keeping the number of parameters fixed (instead of growing linearly with the number of time points). The advantage of using black-box variational inference algorithms is that the framework is highly flexible, allowing for easy modifications and extensions of the underlying generative model without any changes to the inference machinery. This renders the model very promising for clinical applications where generative models might need to be tailored toward specific diseases. A publication on pDCM is currently in preparation and the toolbox will be released in TAPAS was the paper is published.

### Generative Models of Behavioral Data

Neuroimaging provides functional readouts from disease-relevant neural circuits and thus delivers data for models of pathophysiology. However, these data and models are usually not suitable for drawing direct conclusions about cognition and its disturbances. By contrast, behavioral data can be used for inference on an agent's internal processes at the algorithmic (information processing) level [for a review of guidelines for the computational modeling of behavioral data, please refer to (177)]. Importantly, acquisition of behavioral data is often easier, cheaper and more patient-friendly than neuroimaging data, and computational models of behavior thus hold great promise for establishing clinically useful computational assays—on their own or in combination with neuroimaging data (16, 29). At present, TAPAS contains two different generative models of behavioral data which will be discussed next: (i) the Hierarchical Gaussian Filter [HGF; (178)], and (ii) the Stochastic Early Reaction, Inhibition and late Action model [SERIA; (179)].

#### Hierarchical Gaussian Filter (HGF)

The *Hierarchical Gaussian Filter* [HGF; (178)] is a hierarchical Bayesian framework for individual learning under the various kinds of uncertainty which arise in realistic non-linear dynamic systems (e.g., perceptual uncertainty, environmental volatility). Importantly, the hierarchy implemented in the HGF is not to be confused with the hierarchy that was discussed in the context of empirical Bayes and, more specifically, HUGE. While in empirical Bayes, the hierarchy (typically) refers to a multi-subject or mixed-effects structure where the levels represent single-subject and group-level information, the HGF implements a hierarchy in which the levels represent the temporal evolution of latent states. More specifically, it consists of two parts: a generative model, the HGF-GM, which describes the stochastic evolution of the non-linearly coupled hidden states of a dynamic system; and the HGF proper, a set of deterministic update equations resulting from the variational inversion of the HGF-GM. The HGF proper contains the Kalman filter as a special case, but is also suited for filtering inputs generated by non-linear environments. Combined with an observation model, the HGF proper represents a particular implementation of the “observing the observer” framework developed by Daunizeau et al. (220, 221). This framework is based on the separation of two model components: (i) a perceptual model which describes an agent's inference on the environment (in this case the HGF proper), and (ii) a response model which describes how inferred latent states of the agent translate into the agent's observed actions, such as, decisions or responses (180).

**Figure 7:**
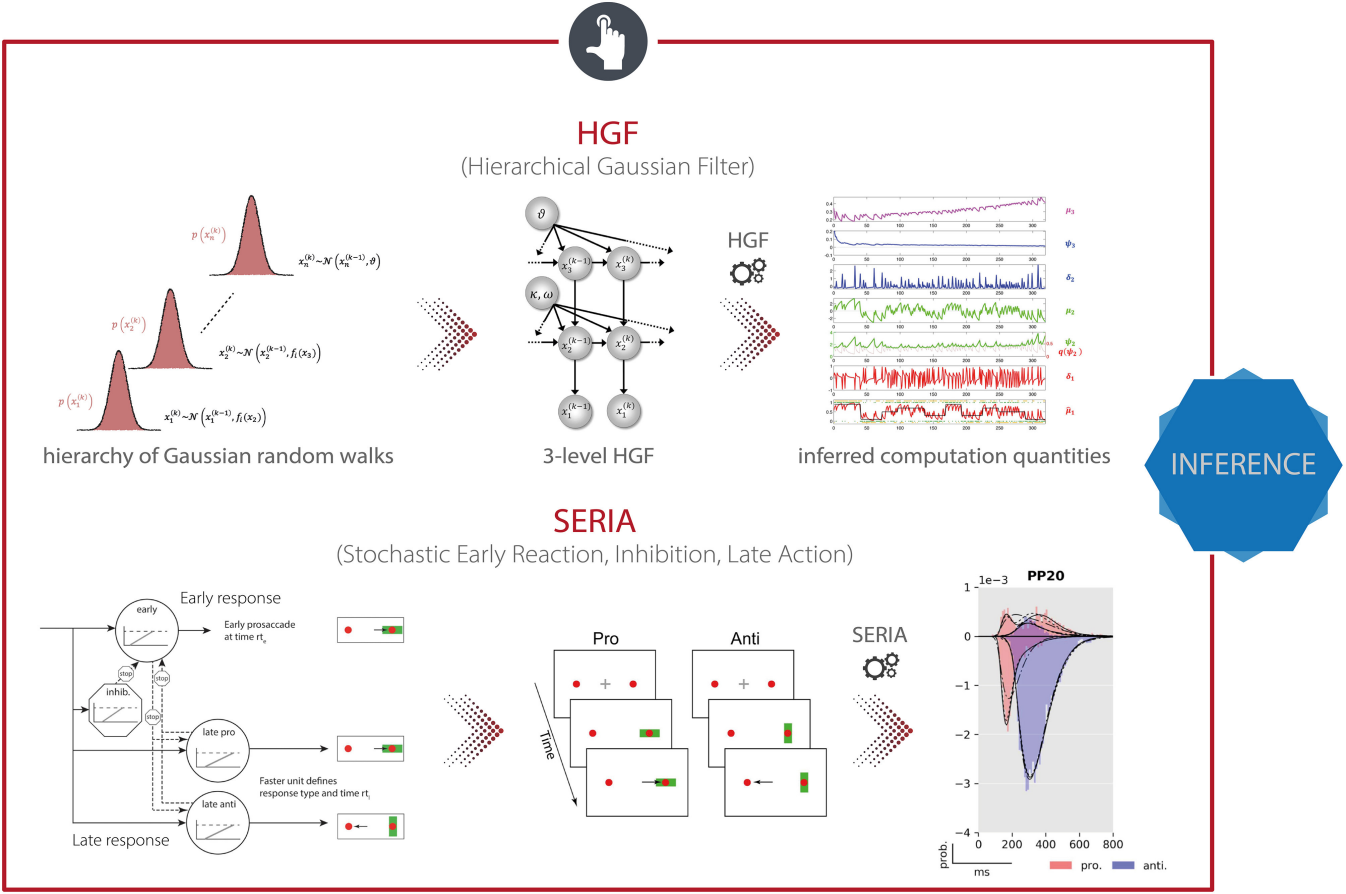
TAPAS components that implement generative models of behavioral data. **(Top)** The Hierarchical Gaussian Filter (HGF) is a hierarchical Bayesian model for individual learning under different forms of uncertainty (e.g., perceptual uncertainty, environmental volatility). **(Bottom)** The Stochastic Early Reaction, Inhibition, and late Action (SERIA) model represents a computational model of an agent's behavior during the antisaccade task by modeling early reflexive and late intentional eye movement via two interacting race-to-threshold processes.

In the HGF, the perceptual model takes the form of a hierarchical Bayesian model where the temporal evolution of states at any level (except the first) are represented as Gaussian random walks or first-order autoregressive processes (Figure 7, *top*). Importantly, the step size of each walk (i.e., the variance of the Gaussian distribution) depends on the state at the next higher level. This coupling between levels is controlled by subject-specific parameters that shape the influence of uncertainty on learning. Under a VB approximation, one can derive efficient trial-by-trial update equations for this model that describe the agent's belief updating. Importantly, these update equations rest on precision-weighted prediction errors (PE) at different levels of the hierarchy. In other words, the HGF tracks an agent's expression of (approximate) Bayesian learning in the presence of uncertainty under the assumption that the brain continuously updates a hierarchical generative model of sensory inputs, with PEs serving as the teaching signal. This perceptual model is then combined with a response model (e.g., unit-square sigmoid or softmax function; although a wide range of different response models is available) that links the agent's current estimates of the latent states to observed actions, such as motor or physiological responses (180). In combination with priors on the model parameters, this specifies a full generative model of observed responses that is inverted using maximum-a-posteriori (MAP) estimation. In summary, the HGF provides a generic approximation to subject-specific instantiations of hierarchical Bayesian learning. This generic form renders the HGF applicable to a wide range of scenarios, including discrete and continuous latent states, deterministic and probabilistic relations between environmental events and latent states, as well as learning under multiple forms of uncertainty such as perceptual uncertainty and environmental volatility (178).

Depending on the specific scientific question, the HGF can (or must) be compared to other models of learning. For example, the HGF toolbox already implements various other models of learning, including the Rescorla-Wagner model (181), Sutton model (182), and a Hidden Markov model (183). These models differ from the HGF in their proposed style of learning. For instance, while the updating in Rescorla-Wagner (RW) learning is structurally not dissimilar to that in the HGF (i.e., weighted prediction errors), the RW model differs fundamentally from the HGF in that prediction errors are weighted by a constant learning rate instead of time-dependent precision weights (178). These (and any other learning models of interest outside the toolbox) can be compared using Bayesian model selection [BMS; (117, 158)]. This is achieved by harvesting (an approximation to) the log model evidence from the different models and entering the estimates into existing tools for fixed-effects or random-effects BMS, e.g., SPM (184) or the VBA toolbox (35).

For applications in TN/CP, parameter estimates of the HGF (or any other learning model in the toolbox) can be used to characterize perceptual inference and decision-making in specific disorders [e.g., (18, 19)]; alternatively, and perhaps even more frequently, the estimated trajectories of precision-weighted PEs are used in trial-by-trial analyses of fMRI and EEG data [e.g., (20, 185, 186)]. The HGF can also be applied to other time series than behavioral ones. In an example from TN/CP, Brazil et al. (219) applied an HGF directly to BOLD signal time series from an fMRI experiment.

#### Stochastic Early Reaction, Inhibition and late Action (SERIA) model

Eye movements represent a potentially very interesting functional readout for TN/CP. In addition to the experimental ease with which many data points can be measured, eye movements are disturbed in numerous psychiatric conditions (187–189). An experimental paradigm that has been used frequently in this context is the antisaccade task (190) where participants are asked to suppress a reactive eye movement toward a visual cue and concurrently perform a saccade in the opposite direction (antisaccade). This task is of relevance for clinical applications since it has been widely used to study psychiatric and neurological diseases (188). Most prominently, the antisaccade task is hampered in schizophrenia (191, 192) where an elevated error rate has been proposed as an intermediate phenotype or endophenotype of the disease (193).

TAPAS comprises a computational model of an agent's behavior during the antisaccade task—the *Stochastic Early Reaction, Inhibition and late Action* (SERIA) model (179). Specifically, in order to model error rates and reaction times during the task, SERIA postulates two interacting processes (Figure 7, *bottom*): (i) a fast GO/NO-GO race between a prepotent response (prosaccade) toward the visual cue and a signal to cancel this erroneous action, and (ii) a slow GO/GO race between two units encoding the cue-action mapping, accounting for slow voluntary saccades. The parameters of this model, which are estimated using a sampling-based hierarchical Bayesian scheme, are sensitive to dopaminergic and cholinergic manipulations and were found to allow for out-of-sample predictions about the drug administered to an individual with 70% accuracy (194).

#### Future Developments

As for the generative models of neuroimaging data, extensions to the behavioral models will also be released as part of TAPAS in the future. In particular, an extension of the HGF is currently under development which aims to embed the classical HGF within an empirical Bayesian (EB) scheme. This hierarchical (in the sense of an EB scheme) version of the HGF combines HGFs at the individual level with a layer that represents group effects. Similar to HUGE, this formulation affords a principled way of estimating (empirical) priors from the data, effectively constraining single-subject estimates by group-level information. Inference in the H2GF rests on sampling-based MCMC methods that provide not only an estimate of individual model parameters, but also an approximation to the model evidence via thermodynamic integration (or other suitable techniques).

## Clinical Application

Computational assays are developed with the goal to solve concrete clinical problems. Here, we briefly consider 3 fundamental problems in psychiatry: (i) differential diagnosis (i.e., differentiation between several plausible conditions/mechanisms underlying a patient's symptoms), (ii) stratification of heterogeneous disorders into subgroups, and (iii) prediction of clinical trajectories or treatment responses (25). Different computational strategies are available to address each of these challenges.

First, differential diagnosis can be formalized as hypothesis testing, which—in a Bayesian framework—is equivalent to Bayesian model selection [BMS; (117, 158)] where different hypotheses (models) are compared in the light of observed neuroimaging and/or behavioral data. Specifically, by formalizing competing pathophysiological and/or psychopathological theories in terms of distinct models, we can assess the relative plausibility of these hypotheses by model comparison (119). This rests on comparing the model evidence, an index of model goodness that trades off accuracy and complexity. One early example of how model comparison can be used to support differential diagnosis was provided by a study on NMDA receptor antibody encephalitis (195). This disorder was only discovered relatively recently (196) and is poorly understood at the level of cortical circuit dysfunction. To disambiguate alternative circuit-level mechanisms how seizure activity may unfold during NMDA receptor antibody encephalitis, Cooray et al. (195) compared different implementations of a cortical microcircuit model (DCM). These alternatives differed in the type of synaptic connections that were allowed to change between seizure and non-seizure periods. Using artifact-free seizure data from two patients, the authors demonstrated that concomitant changes in excitatory and inhibitory connections, as well as the gain of inhibitory neurons, best explained the symptoms (seizure recordings).

Second, prediction of clinical trajectories and treatment outcome as well as stratification of spectrum disorders can be achieved by means of supervised and unsupervised generative embedding [GE; (197)], respectively. In brief, the key idea of GE is to perform (un)supervised learning in a feature space that is spanned by the posterior estimates obtained from a generative model fitted to the data. In the simplest way, this can be achieved by following a two-step procedure: First, a generative model of (neuroimaging or behavioral) data is used to infer the posterior densities over model parameters (e.g., neuronal connectivity, learning rate). Second, summary statistics of these posterior densities (e.g., maximum-a-posteriori estimates) enter a supervised (classification, regression) or unsupervised (clustering) machine learning technique. In doing so, the generative model serves as a theory-driven dimensionality reduction device which projects the high-dimensional and noisy data onto neurobiologically meaningful parameters that span a low-dimensional and interpretable space for (un)supervised learning. GE frequently yields more accurate results than conventional ML (30, 139, 198), likely because the generative model separates signal (reflecting the process of interest) from (measurement) noise. For instance, in a recent study, Frässle et al. (198) utilized GE (combining DCM for fMRI and linear support vector machines) to predict the 2-year clinical trajectories of patients with major depressive disorder (MDD) from the NEtherlands Study of Depression and Anxiety (NESDA). Specifically, using GE, the authors could distinguish chronic patients from fast-remitting patients with 79% balanced accuracy. Similarly, gradually improving patients could be distinguished from fast-remitting patients with 61% balanced accuracy. This significantly outperformed classification based on conventional (descriptive) features, such as local activation or functional connectivity estimates, which were obtained from the same data. These results (in line with other studies) illustrate the potential of GE for clinical decision making. In what follows, we outline toolboxes included in TAPAS that can be used for supervised and unsupervised GE.

### Classification and Prediction

As outlined above, GE is typically implemented in terms of a two-step procedure: (i) generative models of measured data are inverted for each subject individually, and (ii) the summary statistics of the posterior estimates (e.g., the maximum-a-posteriori estimates) are used for supervised (classification, regression) or unsupervised (e.g., clustering) learning.

Here, we first focus on supervised GE as a formal way of performing differential diagnosis (via classification) or outcome prediction. To this end, TAPAS comprises the *Generative Embedding* (GE) toolbox, Python-based software that facilitates the generative embedding framework and allows for exploration and visualization of classification performance. The toolbox is a wrapper around scikit-learn (199), with the goal of providing a set of convenient functions and sensible defaults that form a suitable starting point for generative embedding analyses. The GE toolbox can use posterior parameter estimates from any of the generative models mentioned above as input features, and performs binary or multi-class classification. The toolbox utilizes logistic regression as its default classifier because it represents a simple linear model that protects against overfitting, is (relatively) simple to interpret, and the ensuing class probabilities are useful for interpreting classifier outputs. Furthermore, the toolbox implements a repeated k-fold cross-validation as the default procedure for both model selection (i.e., hyperparameter tuning) and model validation (i.e., estimating out-of-sample performance), including the possibility for within-fold confound correction (200). This choice is motivated by the fact that, in comparison to leave-one-out cross-validation, k-fold cross-validation has a lower variance and is therefore less prone to overfitting (201). Finally, significance testing of classification performance is done by default using permutation tests as they provide an unbiased estimate of error variance. This is in contrast to parametric tests (e.g., binomial confidence intervals, McNemar's test) which typically underestimate variance and are therefore overconfident (202).

### Stratification of Heterogeneous Psychiatric Disorders

The goal of stratifying heterogeneous disorders is to identify subgroups that share common pathophysiological or psychopathological mechanisms. The increased homogeneity in terms of underlying disease mechanisms increases the power of clinical trials and enhances predictions of clinically relevant outcomes (25). One way to achieve this goal is by using posterior parameters from a generative model for unsupervised learning (e.g., clustering). This approach has been utilized by Brodersen et al. (139) to identify distinct subgroups in a heterogeneous cohort of 41 patients with schizophrenia based on effective connectivity patterns during a working memory task (138). The authors showed that these purely physiologically informed and connectivity-based subgroups also differed clinically, as illustrated by significant differences in their negative symptom severity scores on the Positive and Negative Syndrome Scale (PANSS). An alternative to this two-step GE procedure is implemented in HUGE, a toolbox we already discussed in the context of generative models of neuroimaging data (161, 162). Specifically, HUGE casts unsupervised GE as a single hierarchical generative model that simultaneously describes individual data generation and assigns participants to clusters (Figure 5, *bottom*). Unifying these two steps has a couple of conceptual advantages: (i) the hierarchical nature of the model allows learning prior distributions from the data (i.e., empirical Bayes), (ii) model inversion at the single-subject level is regularized by (cluster-specific) group results, and (iii) clustering takes the uncertainty about individual connectivity parameter estimates into account.

## Tapas in Action

Finally, we briefly discuss a few selected examples from previous work that made use of different toolboxes from TAPAS. In particular, here we focus on studies that investigate pathophysiological/pathocomputational mechanisms and/or explore the role of neuromodulatory transmitter systems in these processes. The latter are a particularly prominent topic for clinical applications of generative models because the majority of available pharmacotherapeutic approaches in psychiatry targets synthesis, metabolism or receptors of neuromodulatory transmitters.

In order to non-invasively infer upon the status of neuromodulatory systems (e.g., dopamine, acetylcholine, serotonin, noradrenaline), various studies have combined experimental manipulations of different neuromodulatory systems with generative modeling of neuroimaging or behavioral data. In a first step, Iglesias et al. (185) have provided evidence for hierarchical belief updating during a sensory associative learning task under volatility and without rewards. Hierarchical belief updating via PEs plays a central role in “Bayesian brain” theories, such as predictive coding (170, 203). Iglesias et al. (185) utilized the HGF to infer upon subject-specific trajectories of precision-weighted PEs at different levels of the hierarchy which were then used in a GLM of fMRI data. They found that low-level PEs, encoding the mismatch between prediction and actual visual stimulus outcome, were reflected by widespread BOLD activity in visual and supramodal areas, but also in the midbrain. Conversely, high-level PEs, encoding the mismatch between prediction and actual stimulus probabilities, were reflected by BOLD activity in the basal forebrain. Midbrain and basal forebrain contain dopaminergic and cholinergic neurons, respectively, suggesting that (i) dopaminergic midbrain neurons might signal PEs unrelated to reward and (ii) cholinergic neuron activity in the basal forebrain might reflect PEs about probabilities and may thus relate to “expected uncertainty” (204). Although a subsequent pharmacological study in human volunteers using the same paradigm did not support this notion (205), the finding that midbrain activity may reflect reward-unrelated prediction errors has since been replicated in several animal (206, 207) and human studies (208).

Importantly, neural correlates of computational quantities can not only be detected in fMRI data, but can also be found in EEG signals, where the superior temporal resolution allows for characterizing their precise temporal dynamics. For instance, Weber et al. (186) related HGF estimates to single-trial EEG data from participants who received ketamine in a placebo-controlled, double-blind, within-subject fashion. The authors demonstrated that PE-related activity was found in a temporal order consistent with hierarchical Bayesian theory. Additionally, they observed a significant impact of ketamine on the high-level PE about transition probabilities. Focusing on behavior only, further evidence has been provided for associations between computational quantities and the status of neuromodulatory systems. For instance, Vossel et al. (230) perturbed the cholinergic system using pharmacological interventions, and utilized the HGF to demonstrate that this led to an increase in the rate of belief updating about cue validity during a modified Posner's task. Similarly, Marshall et al. (225) utilized pharmacological interventions in combination with the HGF to characterize the influence of noradrenergic, cholinergic and dopaminergic antagonists on individual estimates of uncertainty during a probabilistic serial reaction time task. The authors identified different roles for the different neuromodulatory systems, linking noradrenaline to unexpected uncertainty, acetylcholine to environmental uncertainty, and dopamine to uncertainty representations for fast, adaptive responses. Finally, Aponte et al. (194) demonstrated that computational quantities sensitive to neuromodulatory processes can also be derived from generative models of reflexive eye movements. Specifically, the authors conducted a double-blind placebo-controlled pharmacological study and found that computational quantities derived from an antisaccade task using the SERIA model can distinguish between dopaminergic and cholinergic effects on action selection and inhibitory control, allowing for out-of-sample predictions about the drug administered with 70% accuracy. In summary, both the HGF and SERIA comprise computational quantities that are sensitive to the functional status of different neuromodulatory systems.

Beyond questions of pathophysiology and pharmacology, tools from TAPAS have also been used to characterize clinical populations. For instance, Powers et al. (19) studied conditioned auditory hallucinations in four groups of people who differed both in their voice-hearing and treatment-seeking statuses. Utilizing the HGF to infer upon the participants' individual beliefs, the authors demonstrated that the weighting of prior beliefs was significantly larger in people with hallucinations than their non-hallucinating counterparts. This is consistent with the hypothesis that, in the context of a Bayesian brain, hallucinations may be explained by overly strong priors (209). Focusing on patients with autism spectrum disorder (ASD), Lawson et al. (18) utilized the HGF to provide evidence that ASD patients tend to overestimate volatility in the face of environmental changes. This leads to reduced learning about unexpected (surprising) events, which might serve as an explanation for the typical insistence on sameness and intolerance of change in ASD patients (210). Finally, Cole et al. (20) applied HGF estimates from an associative learning task to characterize brain responses to precision-weighted PEs in individuals at clinical high risk (CHR) for psychosis. Compared to a healthy control group, CHR individuals showed enhanced PE responses in several (particularly prefrontal) regions, consistent with the prediction from the dysconnection hypothesis of schizophrenia (125, 164) that (proneness to) psychosis is characterized by abnormal precision-weighted PE signaling in cortex. Furthermore, prefrontal PE activity was correlated with clinical status.

TAPAS tools that implement generative models of neuroimaging data have also been applied to clinical populations—although this is still rare. For instance, Yao et al. (162) applied HUGE to an fMRI dataset comprising aphasic patients (with a lesion in the left frontal and/or temporal cortex) and healthy controls (211) for an initial demonstration of the potential clinical utility of the model for patient stratification. In brief, the authors demonstrated that HUGE correctly identifies two clusters in the dataset, which mapped almost perfectly onto aphasic patients and healthy controls, yielding a balanced purity of 95.5%. While it is important to emphasize that diagnosing patients with aphasia does not yet represent a truly meaningful clinical problem, it demonstrates the practical utility of HUGE for stratification in a scenario where ground truth is known. Furthermore, regression DCM has been used to study alterations in whole-brain effective (directed) connectivity between psychotic patients, their first-degree relatives, as well as matched healthy controls. The authors demonstrate that patients showed distinctly different whole-brain connectivity patterns from healthy controls and first-degree relatives, and that the connectivity patterns allow for significant discrimination at the individual level. A publication on this work is currently in preparation.

Overall, the above studies illustrate the potential of generative models of behavioral and neuroimaging data for clinical applications. However, these studies do not yet implement the kind of end-to-end analysis pipeline that we outlined, at the beginning of the article, as a basis for future computational assays. Instead, the above studies simply used selected components from TAPAS at a time. Having said this, recent work by Harrison et al. comes close to the kind of end-to-end pipeline highlighted above, combining multiple components from TAPAS (212). In brief, the authors aimed to investigate interoception and how anxiety relates to the perception of internal bodily states. To this end, Harrison et al. employed two paradigms available in TAPAS Tasks, namely the Filter Detection (FD) and Breathing Learning (BL) task. The FD task revealed differences in sensitivity to breathing perception and altered interoceptive metacognitive bias between low-anxiety and moderate-anxiety healthy controls. Furthermore, for the BL task, the authors acquired fMRI data using a high-field 7T MR scanner. PhysIO was employed for physiological noise correction based on measurements of cardiac and respiratory cycles. fMRI then underwent thorough preprocessing and artifact removal by combining tools from various software packages, including FSL and SPM. Brain activity coupled with dynamic changes in bodily states was then modeled using subject-specific trajectories of predictions and PE which have been inferred utilizing the HGF toolbox. This revealed the anterior insula to be associated with both interoceptive predictions and PEs, where the former was also differentially expressed in the low and moderate anxiety groups.

While this moves toward the kind of end-to-end analysis pipeline that we outline, it is important to note that the two groups tested by Harrison et al. do not represent clinical groups (but were recruited from the healthy population) and the study thus lacks the final module of the aforementioned pipeline (i.e., Clinical Application; see Figure 2). Furthermore, it is important to keep in mind that none of the studies mentioned above is yet of any direct clinical utility, in the sense that they do not address a practical clinical question, such as differential diagnosis or predicting outcomes/clinical trajectories. The latter in particular requires data from prospective studies that include information about future clinical outcomes—a critical condition for validating computational assays (16). Unfortunately, so far, these datasets are rare.

## Conclusion

In this article, we have described the **T**ranslational **A**lgorithms for **P**sychiatry-**A**dvancing **S**cience (TAPAS) software package, an open-source collection of toolboxes (primarily written in MATLAB; with some components in C and Python) that aim to facilitate the acquisition and (computational) analysis of neuroimaging and behavioral data. Specifically, we reviewed the different toolboxes in TAPAS and highlighted how these might support the construction of end-to-end analysis pipelines—from raw data to clinical applications.

## Software Note

The **T**ranslational **A**lgorithms for **P**sychiatry-**A**dvancing **S**cience (TAPAS) software package, comprising all toolboxes described in this paper, is freely available as open-source code (https://www.translationalneuromodeling.org/tapas).

## Author Contributions

SF and KS wrote manuscript. All authors helped revising the manuscript. Furthermore, several authors contributed software code to the TAPAS software package reviewed in this manuscript.

## Conflict of Interest

The authors declare that the research was conducted in the absence of any commercial or financial relationships that could be construed as a potential conflict of interest.

## Footnotes

^1^ML is also used “on its own” in CP and applied directly to measured data, e.g., for producing patient-specific predictions or discovering structure in heterogeneous populations (222–224, 226–228). In this paper, however, we focus on approaches where ML operates on estimates provided by generative models.

^2^Exteroception refers to perception of sensations originating from the external world, whereas interoception refers to perception of sensations originating from the own body or “internal world”.
